# Development of 3D neuromuscular bioactuators

**DOI:** 10.1063/1.5134477

**Published:** 2020-03-10

**Authors:** Onur Aydin, Austin P. Passaro, Mohamed Elhebeary, Gelson J. Pagan-Diaz, Anthony Fan, Sittinon Nuethong, Rashid Bashir, Steven L. Stice, M. Taher A. Saif

**Affiliations:** 1 Department of Mechanical Science and Engineering, University of Illinois at Urbana-Champaign, Urbana, Illinois 61801, USA; 2 Regenerative Bioscience Center, University of Georgia, Athens, Georgia 30602, USA; 3 Biomedical and Health Sciences Institute, Division of Neuroscience, University of Georgia, Athens, Georgia 30602, USA; 4 Department of Bioengineering, University of Illinois at Urbana-Champaign, Urbana, Illinois 61801, USA; 5 College of Agricultural and Environmental Sciences, Department of Animal and Dairy Science, University of Georgia, Athens, Georgia 30602, USA

## Abstract

Neuronal control of skeletal muscle bioactuators represents a critical milestone toward the realization of future biohybrid machines that may generate complex motor patterns and autonomously navigate through their environment. Animals achieve these feats using neural networks that generate robust firing patterns and coordinate muscle activity through neuromuscular units. Here, we designed a versatile 3D neuron-muscle co-culture platform to serve as a test-bed for neuromuscular bioactuators. We used our platform in conjunction with microelectrode array electrophysiology to study the roles of synergistic interactions in the co-development of neural networks and muscle tissues. Our platform design enables co-culture of a neuronal cluster with up to four target muscle actuators, as well as quantification of muscle contraction forces. Using engineered muscle tissue targets, we first demonstrated the formation of functional neuromuscular bioactuators. We then investigated possible roles of long-range interactions in neuronal outgrowth patterns and observed preferential outgrowth toward muscles compared to the acellular matrix or fibroblasts, indicating muscle-specific chemotactic cues acting on motor neurons. Next, we showed that co-cultured muscle strips exhibited significantly higher spontaneous contractility as well as improved sarcomere assembly compared to muscles cultured alone. Finally, we performed microelectrode array measurements on neuronal cultures, which revealed that muscle-conditioned medium enhances overall neural firing rates and the emergence of synchronous bursting patterns. Overall, our study illustrates the significance of neuron-muscle cross talk for the *in vitro* development of neuromuscular bioactuators.

## INTRODUCTION

Biohybrid machines that utilize muscle cells to actuate compliant artificial scaffolds are emerging as novel platforms for bioengineering and soft robotics applications (see the study by Ricotti *et al.*^1^ for a recent review). Several biohybrid machines capable of untethered locomotion have been developed over the past decade, using cardiac^2–6^ or skeletal^7–9^ muscle cells. More recently, a biohybrid swimmer actuated by neuromuscular units has been demonstrated whereby contractions of an engineered skeletal muscle tissue were evoked by stimulation of co-cultured motor neurons (MNs) on a free-standing scaffold.^10^ Since animals use their nervous system to orchestrate complex motor patterns and adaptively respond to their environment, neuronal control of skeletal muscle bioactuators could potentially enable the development of future biohybrid machines capable of exhibiting the high-level locomotor behaviors observed in animals. Advances toward this goal currently require a deeper understanding of neuromuscular development.

A high degree of cross talk between neurons and muscles comes into play during the development of neuromuscular units.^11–13^ Such cross talk entails reciprocal biochemical and biophysical interactions, which can be dependent upon spontaneous activity or be mediated by soluble factors. These bidirectional interactions are thought to play key roles in attaining neuromuscular units with a proper form and function.^11–16^ Furthermore, in vertebrates, MNs in the spinal cord participate in neural networks^17^ where interactions among large populations of neurons lead to robust and coordinated firing activity typically in the form of synchronous bursting patterns.^18,19^ The design of next-generation neuromuscular bioactuators therefore warrants an investigation of how muscles and neural networks may co-develop in a biohybrid setting.

To address this question, we demonstrate here a 3D neuron-muscle co-culture platform to serve as a test-bed for neuromuscular bioactuator development. The platform architecture allows co-culture of a neuronal cluster with up to four separate target tissues. The targets comprise free-standing engineered tissue constructs that are anchored by compliant pillars, which allow the measurement of contraction forces. First, we co-cultured optogenetic mouse embryonic stem cell (mESC)-derived neurospheres containing MNs with skeletal muscle strips as target tissues and verified the formation of functional neuromuscular junctions (NMJs). We then demonstrated the effects of target-specific long-range interactions on neuronal outgrowth patterns by using muscles, fibroblasts, and acellular matrix as targets. Next, we showed that muscles co-cultured with neurons exhibit a significant increase in spontaneous contractility and a correspondingly higher degree of sarcomere assembly following NMJ formation compared to muscles in mono-culture. Finally, we performed a separate microelectrode array (MEA) electrophysiology assay to investigate neural network activity. We compared firing patterns of networks cultured in regular medium with those cultured in muscle conditioned medium (CM). Our results revealed that muscle CM significantly enhances the overall bursting rate as well as the emergence of synchronous bursting of neural networks. Taken together, our results illustrate improved functional outcomes of both muscles and neural networks as they co-develop, and our platform provides an avenue for further inquiry into the roles of synergistic interactions in neuromuscular bioactuator development.

## RESULTS AND DISCUSSION

### Multi-target 3D neuron-muscle co-culture platform

**FIG. 1.**
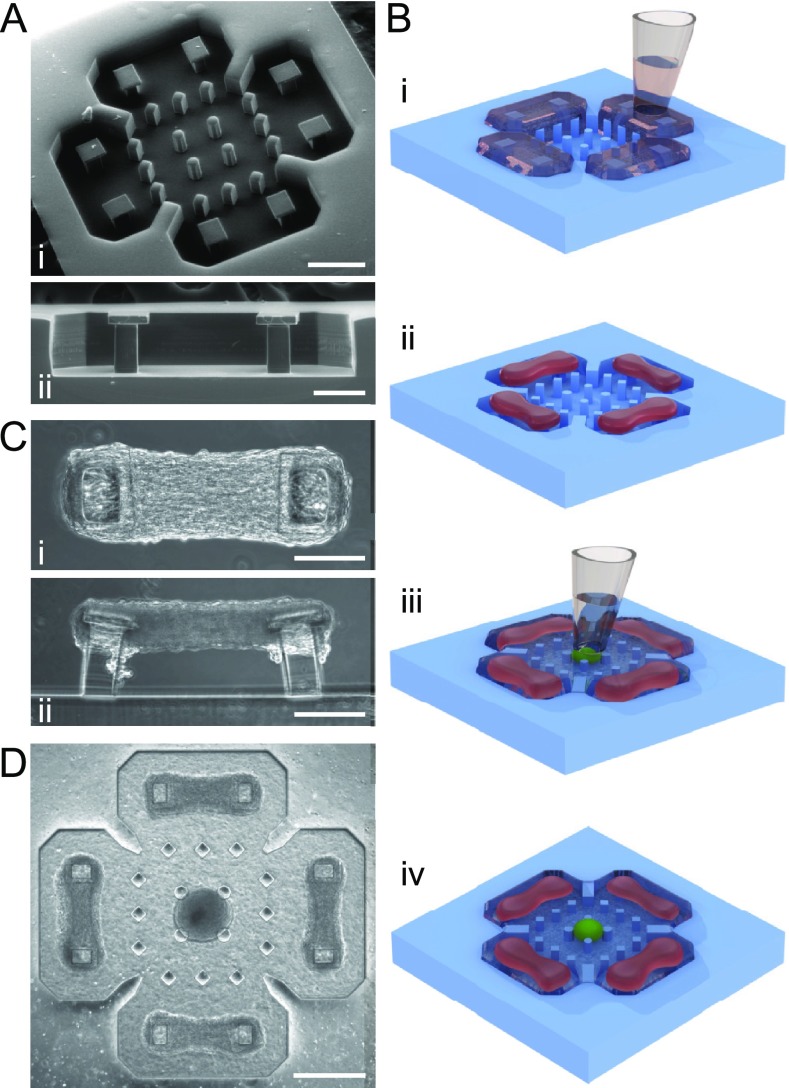
3D co-culture in the engineered platform. (a) SEM images of (i) the full platform and (ii) one of the target wells showing the pillar profile. (b) Schematic of the tissue seeding process illustrating (i) target cell-ECM seeding, (ii) compacted target tissues, (iii) neurosphere seeding, and (iv) co-culture tissue. (c) Phase contrast images of a muscle strip from (i) top and (ii) side views. (d) Phase contrast image of the full platform after neurosphere seeding. Scale bars: [(a-i) and (d)] 500 *μ*m and [(a-ii), (c-i), and (c-ii)] 200 *μ*m.

We designed and microfabricated a polydimethylsiloxane (PDMS) platform for multi-target neuron-muscle co-culture in a 3D and compartmentalized setting. The platform has a central compartment to host a neurosphere surrounded by four target compartments each containing a pair of T-shaped pillars to anchor the target tissues and measure contraction forces [Fig. 1(a)]. All compartments have a nominal depth of 200 *μ*m, enabling 3D culture settings while still allowing visualization of tissues under a light microscope. Neuron-muscle co-culture is achieved in two stages [Fig. 1(b)]: first, the target muscle tissues are formed by mixing skeletal myoblasts with an extracellular matrix (ECM) solution consisting of type I collagen and Matrigel and seeding the mixture directly into the target wells by the pipette. The muscle tissues are anchored by the two compliant pillars [Fig. 1(c)], which allows us to quantify muscle contraction forces by optically measuring pillar deflections. In the second stage, a neurosphere containing motor neurons is seeded in the center and the entire platform is filled with ECM [Fig. 1(d)].

The simple approach of seeding tissues directly by pipetting makes it possible to design a multi-target platform, which improves the experimental yield by increasing the number of target tissues per neuronal cluster. This multi-target architecture may also allow our platform to serve as a useful test-bed for modeling bioactuators that employ multiple muscle tissues such as the system recently demonstrated by Morimoto *et al.*, which involved a pair of antagonistic muscles on a compliant scaffold.^20^ Furthermore, the present platform design provides about 500 *μ*m separation between the neurosphere and target tissues [Fig. 1(d)], thus allowing the use of the platform to investigate long-range interactions. This compartmentalization is enabled by the hydrophobicity of PDMS: In each target well, the side that faces the central compartment has three posts separated by small (120 *μ*m) gaps. When the liquid cell-ECM mixture is seeded only into the target wells, hydrophobicity of PDMS induces the formation of menisci between the posts,^21^ preventing the liquid cell-ECM mixture from leaking into the central compartment (Fig. S1). After the compaction of cell-laden ECM gels into tissues inside the four target wells, the neurosphere is then seeded in the center and all five compartments are filled with ECM, thereby creating a continuous yet compartmentalized co-culture. Moreover, the PDMS posts that surround the neurosphere and the muscle compartments may also help maintain compartmentalization throughout co-culture since confinement by PDMS posts has been shown to reduce the collective migration rates of 3D multicellular tissue constructs.^22^

### Characterization of neuromuscular bioactuators

We developed co-cultures in our platform using C2C12 mouse skeletal myoblasts to create target muscle strips and co-culture them with neurospheres that were obtained by directed differentiation of an optogenetic mESC line toward motor neurons (see Methods). After initiating co-culture, we observed neuronal outgrowth during the first 2–3 days. We then monitored co-cultures and performed optical stimulation to assess the formation of NMJs. In all optical stimulation assays, the entire field of view was illuminated with blue light for 1 s while continuously recording the video of the sample to capture muscle activity before, during, and after stimulation. To confirm that illuminating the entire sample does not lead to unintended stimulation of muscle strips, we prepared muscle-only control samples where muscle strips were formed and embedded in 3D ECM gel without a neurosphere. Muscle activity was quantified in terms of the contraction force produced by the muscle strips.

**FIG. 2.**
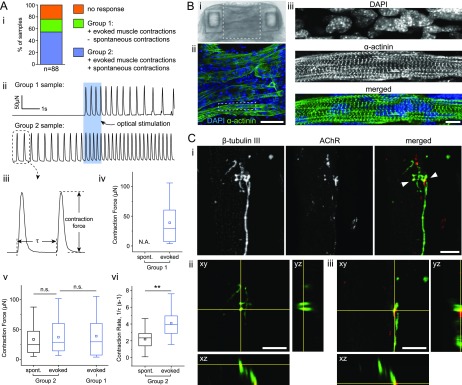
Formation of functional neuromuscular junctions. (a) (i) Muscle strips categorized by their contraction pattern in response to optical stimulation of neurons. (ii) Force-time traces of representative samples from groups 1 and 2. The blue rectangle indicates the optical stimulation. (iii) Definitions of contraction force and period. (iv) and (v) Comparison of spontaneous and evoked contraction forces in groups 1 and 2. (vi) Comparison of spontaneous and evoked contraction rates in group 2. The values in panels iv, v, and vi are averaged over the ten contractions immediately preceding and following optical stimulation for spontaneous and evoked contractions, respectively. Box plots represent the 25th, 50th, and 75th percentiles with whiskers representing 1.5×IQR, n = 19 muscle strips for group 1, n = 48 muscle strips for group 2, and ^**^p < 0.005 (student's t-test). (b) (i) Brightfield image of a muscle strip and (ii) confocal image of the region outlined in (i) illustrating muscle fibers. (iii) Zoomed views of the region outlined in (ii) showing a muscle fiber with cross-striations. (c) (i) Confocal images illustrating a neurite (β-tubulin III, green) extending toward and making connections with post-synaptic receptor clusters on the muscle (AChR, red). (ii) and (iii) Orthogonal views of the regions indicated by the arrowheads in the rightmost panel in (c-i). Scale bars: (b-i) 100 *μ*m and [(b-ii) and (c)] 10 *μ*m.

We began to observe muscle contractions in response to optical stimulation of MNs around day 4–5 of co-culture, corresponding to about 2 days after neurites reach the target muscle strips. In terms of the NMJ formation timeline, this is in agreement with the previously reported results using the same cell sources.^23^ Interestingly, in addition to contractions evoked by stimulation of MNs, some muscle strips also developed rhythmic spontaneous contraction patterns that were present before neuronal stimulation (Movie S1). By day 7, we identified three different muscle behaviors in response to optical stimulation of neurons [Fig. 2(a-i)]: no change in the contraction pattern (21/88 muscle strips), evoked contractions in muscles that were quiescent before stimulation (19/88 muscle strips), and evoked as well as spontaneous contractions (48/88 muscle strips). The presence of evoked muscle contractions in the latter two groups suggests the formation of functional NMJs, and these groups comprised 76% of the muscle strips. For further analysis, we refer to muscles that are quiescent before stimulation as group 1 and muscles spontaneously contracting before stimulation as group 2 [Fig. 2(a-ii)].

To quantify the effect of optical stimulation, we analyzed the muscle contraction dynamics. For each muscle strip, we computed the force and contraction rate averaged over the ten contractions immediately before and the ten immediately after stimulation. Here, we define the contraction rate as 1/τ, where τ is the contraction period measured as the time between two consecutive contractions [Fig. 2(a-iii)]. In group 1, the effect of neuronal stimulation is self-evident since muscle strips are quiescent before stimulation and produce contractions with a force of 39.0 ± 34.4 *μ*N upon neuronal stimulation [Fig.2(a-iv)]. In group 2, there was no significant difference in contraction force before and after stimulation. There was also no significant difference in the strength of evoked contractions in groups 1 and 2 [Fig. 2(a-v)]. However, the evoked contractions in group 2 can be quantitatively distinguished from spontaneous contractions by their significantly higher rate [Fig. 2(a-vi)]. In addition, we observed no effect of light on the contraction dynamics in muscle-only control samples, indicating that contractions evoked by optical stimulation in co-culture samples are due to the formation of NMJs between muscle strips and MNs.

Furthermore, we performed immunofluorescence assay to confirm the morphology of neuromuscular units. The C2C12 myoblasts embedded in 3D ECM had differentiated to form multinucleated muscle fibers with cross-striations [Fig. 2(b)], and neurites extended toward the fibers and made physical connections with post-synaptic acetylcholine receptor clusters [Fig. 2(c)]. Taken together, these results illustrate the formation of optically excitable functional NMJs between MNs in stem cell-derived neurospheres and engineered skeletal muscle tissue constructs in our co-culture platform.

### Neuronal outgrowth toward different targets

**FIG. 3.**
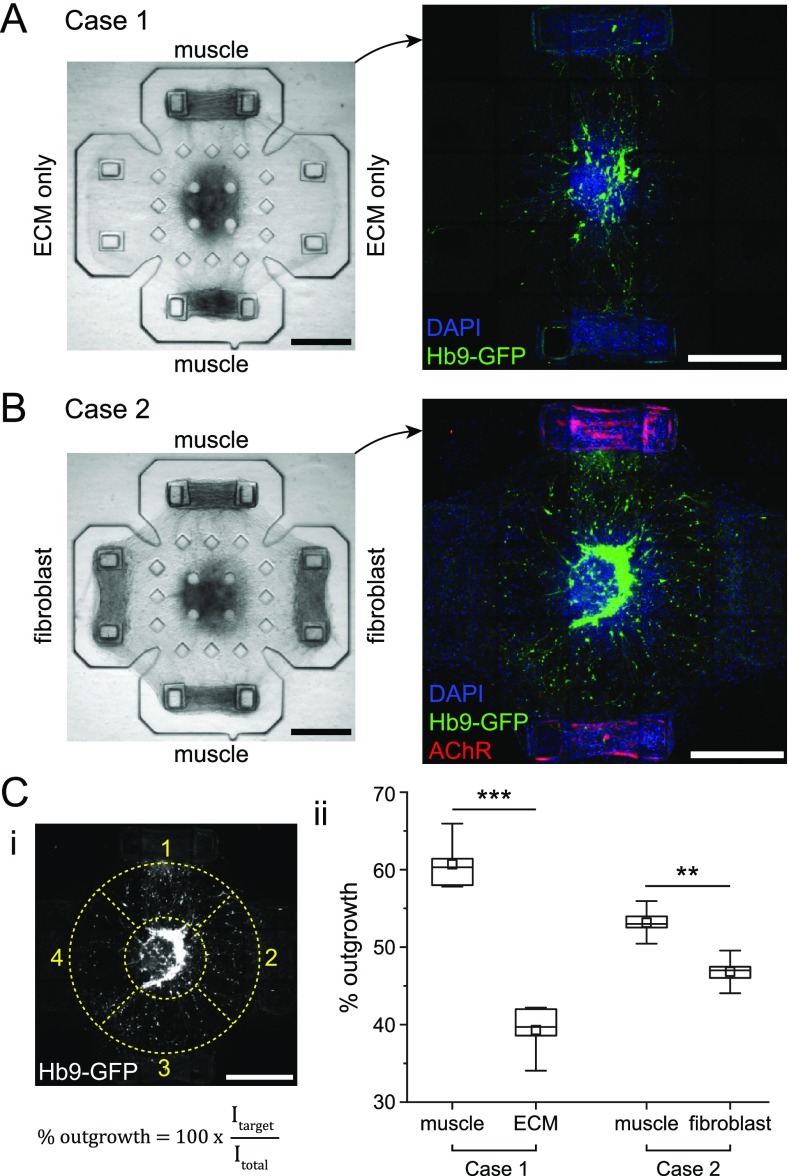
Neuronal outgrowth toward different targets. Brightfield and corresponding confocal images of representative samples from (a) case 1 and (b) case 2. (c) (i) Confocal image of motor neurons with the outlines in dashed lines illustrating the four different sectors that correspond to the regions between the neurosphere and the different targets and the definition of % outgrowth where “I” refers to the total fluorescence light intensity. (ii) Comparison of relative outgrowth toward the different targets in cases 1 and 2. Values are % outgrowth toward each type of target, and box plots represent the 25th, 50th, and 75th percentiles with whiskers representing 1.5×IQR, n = 5 co-culture samples each for cases 1 and 2, ^**^p < 0.005, and ^**^p < 0.0005 (student's t-test). All scale bars: 500 *μ*m.

In the formation of neuromuscular units discussed above, we observed neuronal outgrowth toward the muscle tissues during the first 2–3 days of co-culture. There has been experimental evidence suggesting that soluble factors secreted by muscles promote neuronal outgrowth from MNs in both isolated cultures^24^ and co-cultures.^25^ To investigate if such an effect is present in our co-culture platform, we capitalized on the multi-target and compartmentalized design of the platform and cultured neurospheres with different targets. We performed two sets of experiments where the targets were muscle tissues and acellular ECM in case 1 [Fig. 3(a)] and muscle tissues and fibroblast tissues in case 2 [Fig. 3(b)]. We allowed the co-cultures 3 days and then took fluorescence microscopy images of Hb9-GFP^+^ MNs to visualize outgrowth. To compare outgrowth toward different targets, we draw an annulus centered around the neurosphere, divide it into four sectors corresponding to the regions between the neurosphere and each target, and for each sector quantify the ratio of total fluorescence light intensity in that sector to the total intensity in the entire annulus as a measure of the relative degree of outgrowth toward that target [Fig. 3(c-i)].

In case 1, we observed significantly more outgrowth toward muscle tissues compared to acellular ECM. While this result does support the idea that the presence of muscles can enhance neuronal outgrowth, the mechanism of interaction is ambiguous in this case: As engineered tissue constructs form, due to compaction and remodeling of the ECM, their rigidity increases from initial levels comparable to that of acellular ECM to as much as an order of magnitude higher values.^26^ This can lead to muscle strips and acellular ECM, providing different mechanical cues, and therefore makes it difficult to ascertain whether the biased outgrowth is due to mechanical or biochemical signals. To resolve this issue, we performed the experiments in case 2 where the alternate targets were fibroblast tissues. Fibroblasts embedded in ECM generate compaction and remodeling of the ECM,^27^ the same as muscles, thus eliminating the asymmetry in mechanical cues. Analysis of the case 2 samples also revealed biased outgrowth toward muscles [Fig. 3(c-ii)], suggesting that neuronal outgrowth toward muscles may indeed be enhanced due to muscle-specific soluble factors.

### Co-development of muscle tissues and neural networks through bi-directional interactions

**FIG. 4.**
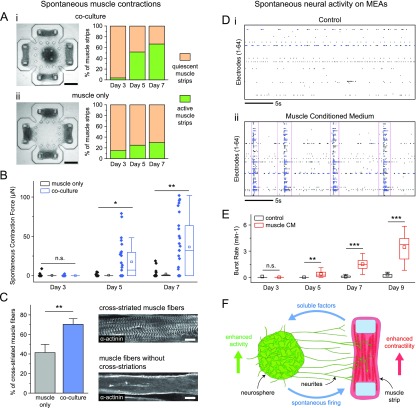
Bidirectional cross talk in developing co-cultures. (a) Representative brightfield images and overall time course of the ratio of active vs quiescent muscle strips in (i) co-cultures and (ii) muscle-only cultures. (b) Spontaneous contraction forces in muscle-only and co-culture samples at days 3, 5, and 7. Values are spontaneous contraction force averaged over a 30 s recording per muscle strip at each day, and box plots represent the 25th, 50th, and 75th percentiles with whiskers representing 1.5×IQR, n = 27 muscle strips for co-culture, n = 20 muscle strips for muscle-only at each day, ^*^p < 0.05, and ^**^p < 0.005 (Mann Whitney U test). (c) Comparison of the percentage of cross-striated muscle fibers between muscle-only and co-culture groups. Bars represent mean ± SD, n = 6 muscle strips for each group, and ^**^p < 0.005 (Mann Whitney U test). Confocal images at the right show sample muscle fibers with and without cross-striations. (d) MEA raster plots of representative samples from (i) control and (ii) CM groups at day 9. Black dashed lines represent the individual spikes, blue dashed lines represent the bursts, and pink boxes outline the synchronous bursts. (e) Time evolution of the MEA burst rate of neurons in control and muscle CM groups. Box plots represent the 25th, 50th, and 75th percentiles with whiskers representing 1.5×IQR, the values are average burst rates per electrode over 10 min recording from the entire well, n = 12 wells each for control and CM at each day, ^**^p < 0.005, and ^***^p < 0.0005 (student's t-test). (f) Conceptual illustration of bidirectional cross talk and its functional outcomes. Scale bars: (a) 500 *μ*m and (c) 10 *μ*m.

During the development of neuromuscular units in our platform, we observed an emergence of spontaneous contraction patterns in a majority of samples [see Figs. 2(a-i) and 2(a-ii)]. Developing skeletal muscle fibers can exhibit spontaneous action potentials and corresponding contractions even in the absence of neurons^28^ possibly due to self-activation of acetylcholine receptors (AChRs) by endogenous secretion of acetylcholine (ACh).^29^ However, the abundance of spontaneous contractions in our co-cultures prompted us to ask whether they may also be neural induced. To investigate, we monitored the spontaneous contractions of muscle strips cultured alone and those co-cultured with neurospheres in our platform. Activity of muscles from both groups was recorded at days 3, 5, and 7 without external stimulation. In co-cultures, the relative number of muscle strips that exhibited spontaneous contractions (i.e., active muscle strips) began to increase at day 5, reaching 67% at day 7 [Fig. 4(a-i)], whereas in the muscle only group, the relative number of active muscle strips remained below 30% [Fig. 4(a-ii)].

In addition to overall contractile activity, we measured and compared the force magnitude of spontaneous contractions [Fig. 4(b)]. At day 3, muscles in both groups either were quiescent or had relatively weak spontaneous contractions with no significant difference in magnitude between muscle-only and co-culture samples. However, starting at day 5, the co-cultured muscle strips had significantly higher spontaneous contraction force compared to the muscle-only group. By day 7, spontaneous contraction forces as high as 102 *μ*N were recorded in co-culture, whereas the maximum spontaneous contraction force in the muscle-only group was 18.8 *μ*N [Fig. 4(b) and Movie S2]. Given this significantly higher spontaneous contraction force in co-cultured muscle strips, we performed immunofluorescence staining of sarcomeric α-actinin on muscle strips from both groups at day 7. In each muscle strip, we observed cross-striated muscle fibers as well as fibers without cross-striations. The relative number of cross-striated muscle fibers was significantly higher in co-cultured muscle strips (70 ± 8%, n = 6 muscle strips) compared to muscle-only (41 ± 6%, n = 6 muscle strips) [Fig. 4(c)], indicating that muscles in co-culture had a relatively high degree of contractile apparatus assembly. This offers a possible explanation for the measurement of higher spontaneous contraction force in co-cultures.

These results prompt the question of how co-culture with neurons could induce higher levels of spontaneous muscle contractility. Previous *in vivo* and *ex vivo* studies on neuromuscular units have shown that MNs spontaneously secrete ACh during development,^30–32^ that there is a marked increase in spontaneous neural activity shortly after neurons come into contact with muscles,^33^ and that this activity can induce contractions in the innervated muscle.^34^ Furthermore, recent *in vivo* work has demonstrated that spontaneous muscle contractions precede and contribute to sarcomere assembly.^35^ Spontaneous firing of neurons and corresponding synaptic transmission is therefore a likely explanation of how the muscles in co-culture in our platform develop more and stronger spontaneous contractions and the associated increase in contractile apparatus assembly. This is further supported by the observation that in our platform, the effect of NMJ formation (i.e., muscle contractions in response to optical stimulation of MNs) begins to appear around day 4–5 of co-culture and that the significant increase in spontaneous muscle contractility also begins at day 5.

To test if our stem cell-derived neurons can develop spontaneous firing patterns, we used microelectrode arrays (MEAs). In MEA electrophysiology of developing neural cultures, the following spontaneous activity pattern can typically be observed: First, cells begin firing randomly, with spikes corresponding to single action potentials observed scattered throughout the MEA. Cells then begin firing in bursts or trains of spikes. Finally, as neural connectivity increases, the neural networks begin to fire in synchronized bursts.^36–38^ We dissociated our neurospheres at the last day of neural differentiation (corresponding to day 0 of co-culture in the 3D NMJ platform), plated them on MEAs at high cell density, and recorded electrical activity over time. After 9 days in culture, we observed spiking and bursting activity in a few electrodes, indicating spontaneous neural activity, but the activity level across the entire culture was relatively low as most electrodes were quiescent [Fig. 4(d-i)]. This is not surprising since it has been shown that with stem cell-derived neurons, the development of robust bursting activity can take several weeks.^39,40^

However, considering the fact that spontaneous neural activity during *in vivo* NMJ development increases markedly shortly after neurons come into contact with muscles,^33^ we postulated that muscle-secreted factors may enhance neural firing. To test this, we cultured neurons on MEAs in muscle-conditioned medium (CM, collected from separate C2C12 cultures) to emulate the postulated soluble factor-mediated retrograde signaling. Strikingly, neurons cultured in muscle CM had substantially improved spontaneous activity [Fig. 4(d-ii)], with robust synchronous busts appearing as early as day 7 in culture (Fig. S2). Quantitatively, the effect of muscle CM corresponded to increased bursting rates with the difference to the control group becoming significant starting at day 5 [Fig. 4(e)]. In terms of the timeline, this matches closely with the increase in muscle contractility that we observed in co-cultures starting around day 5.

## CONCLUSION

We have developed a relatively simple yet versatile 3D co-culture platform as a test-bed for neuromuscular bioactuator development. Using our platform in conjunction with MEA electrophysiology, we investigated the roles of activity-dependent and soluble factor-mediated reciprocal interactions in the co-development of muscle tissues and neural networks. Taken together, our results illustrate synergistic outcomes of neuron-muscle interactions during *in vitro* neuromuscular development [Fig. 4(f)]: muscles secrete soluble factors that enhance spontaneous neural firing and the development of neural networks with synchronous bursting patterns. Neural firing in turn facilitates muscle contractility and the corresponding maturation of contractile apparatus. Our findings illustrate the potential value of identifying mechanisms for modulating these reciprocal interactions for the development of future neuromuscular bioactuators that can achieve predictable and tunable motor patterns.

## METHODS

### Cell culture

C2C12 skeletal myoblasts and NIH/3T3 fibroblasts (both from ATCC) were maintained below 70% confluency in growth medium consisting of high-glucose Dulbecco's modified Eagle's medium (DMEM), 10% v/v fetal bovine serum (FBS), and 2 mM L-glutamine. To facilitate myotube formation by C2C12, they were cultured in muscle differentiation medium consisting of high-glucose DMEM, 10% v/v horse serum, and 2 mM L-glutamine (all reagents from Gibco). All C2C12 and NIH/3T3 cells were used at passage number 5. The optogenetic mouse ESC line ChR2^H134R^-HBG3 Hb9-GFP,^23^ a generous gift from Professor Roger Kamm's lab, Massachusetts Institute of Technology, MA, was maintained in an undifferentiated state on a feeder layer of CF-1 mouse embryonic fibroblasts (Applied Stem Cell) in growth medium consisting of EmbryoMax DMEM (EMD Millipore), 15% v/v ESC-qualified FBS (Gibco), 1X Nonessential Amino Acids (Gibco), 1X EmbryoMax nucleosides (EMD Millipore), 2 mM L-glutamine (Gibco), 0.1 mM β-mercaptoethanol (Gibco), and 10^3^ units/ml leukemia inhibitory factor (EMD Millipore). Neurospheres with MNs were obtained by differentiating ESCs using an established protocol.^41^ On day −6 (i.e*.,* 6 days before initiation of co-culture), ESCs were plated in a tissue culture dish in neural differentiation medium (NDM) consisting of Advanced DMEM/F-12 and Neurobasal medium at a volume ratio of 1:1, 10% v/v KnockOut serum replacement, 2 mM L-glutamine, and 0.1 mM β-mercaptoethanol (all from Gibco). Cells were allowed to aggregate into embryoid bodies (EBs) in NDM for 2 days. On day -4, floating aggregates were collected and plated in a new dish in NDM supplemented with 1 *μ*M retinoic acid (RA) (Sigma) and 1 *μ*M sonic hedgehog agonist purmorphamine (PM) (EMD Millipore) to direct differentiation to MNs. Cells were allowed to differentiate for 3 days. On day −1, EBs were collected and re-plated in NDM supplemented with fresh 1 *μ*M RA and 1 *μ*M PM, as well as 10 ng/ml glial derived neurotrophic factor (GDNF) (Neuromics) and 10 ng/ml ciliary neurotrophic factor (CNTF) (Sigma). On day 0, EBs (neurospheres) were collected and seeded either into the PDMS platform for co-culture or dissociated and seeded onto MEAs for electrical recordings in NDM supplemented with GDNF and CNTF at 10 ng/ml each. The work presented here was based on the use of mouse cell lines mentioned above and did not involve any human subjects, human materials, or live animals. Therefore, there was no requirement for institutional ethics approval for this study.

### PDMS platform fabrication

PDMS platforms were fabricated using microfabricated silicon molds and manual post-processing (Fig. S3). Silicon wafers were patterned by photolithography, etched using the Bosch process, and subsequently coated with polytetrafluoroethylene to facilitate removal of PDMS from the mold. PDMS (Sylgard 184) base and cross-linker were mixed at a ratio of 10:1 by weight, poured onto the silicon molds, and degassed using a vacuum desiccator. Samples were cured at 60 °C for 12 h and peeled off the silicon mold. To achieve the T-shape of the pillars, which is necessary to anchor muscle strips, we attached caps onto the pillars, using a process adapted from previously published studies.^23,42^ Approximately 200 × 150 *μ*m pieces were cut from spin-coated PDMS films of 30 *μ*m nominal thickness using a razor blade mounted on an xyz stage. These caps were then manually glued onto the pillars using uncured PDMS.

### Tissue seeding in PDMS platforms

Prior to tissue seeding, platforms were cleaned by first sonicating in ethanol for 20 min and then autoclaving at 121 °C for 45 min while immersed in DI water. Platforms were then blow dried and sterilized by autoclaving at 121 °C for another 45 min with a drying time of 30 min. For all tissue seeding procedures, ECM solution was prepared on ice by first neutralizing type I collagen from the rat tail (Corning) with 1 N sodium hydroxide, 10× phosphate buffered saline (PBS), and molecular biology grade water and then mixing neutralized collagen thoroughly with growth factor reduced Matrigel (Corning). Collagen and Matrigel were used at final concentrations of 2 mg/ml each. To form muscle or fibroblast strips, C2C12 or NIH/3T3 were suspended in ECM solution at a density of 2.5 × 106 cells/ml. Approximately 0.2 *μ*l of cell-ECM mixture was pipetted into each target well and polymerized at room temperature for 30 min. Samples were then inundated in growth medium and incubated for 1 day, while they compacted the ECM gel and formed a strip. After 1 day, culture medium was switched to muscle differentiation medium to facilitate myotube formation in muscle strips. Samples were kept in muscle differentiation medium for 6 days with daily medium replacements. To initiate neuron-muscle co-culture, medium was aspirated, the entire platform was filled with a fresh ECM solution, and a neurosphere with a diameter of 300–400 *μ*m was manually pipetted into the central well. ECM solution was then allowed to polymerize at room temperature for 30 min. Samples were incubated in NDM with GDNF and CNTF at 10 ng/ml each, with daily medium replacements until experiments were terminated.

### Image acquisition and optical stimulation

All live imaging was performed on an Olympus IX81 inverted microscope (Olympus America) with a digital CMOS camera (Hamamatsu), mounted on a vibration isolation table. The microscope was equipped with an environmental chamber to maintain samples at 37 °C and 5% CO_2_ during imaging. For muscle contraction assays, phase contrast images were taken at 100 fps using a 4× air objective to have the entire sample in the field of view. For optical stimulation, a GFP filter coupled to an X-Cite 120PC Q widefield fluorescent light source (Excelitas Technologies) was used to deliver blue light with a wavelength of 470 nm at 3.9 mW/mm^2^ as measured using a power meter at the sample plane. Samples were stimulated with a 1-second-long bout of light by controlling the motorized shutter of the fluorescent light source.

### Muscle strip force measurement

Pillar deflections caused by muscle contractions were measured from video recordings using the image analysis software Tracker (http://physlets.org/tracker). To compute contraction force, the measured deflections were multiplied by pillar stiffness. Pillar stiffness was estimated using a finite element model of the PDMS pillar created in Comsol Multiphysics (Fig. S4). The elastic modulus of PDMS was measured by nano-indentation to be 1.72 ± 0.14 MPa (mean ±SD, n = 6). The pillar width, thickness, height, and cap height were measured by optical microscopy to be 128.9 ± 1.1 *μ*m, 90.0 ± 1.2 *μ*m, 192.7 ± 5.1 *μ*m, and 26.6 ± 2.1 *μ*m, respectively (mean ± SD, n = 30). A linear elastic constitutive model was used with an elastic modulus of 1.72 MPa and Poisson's ratio of 0.5. Side views of the muscle strips show that muscles wraparound the pillar caps. Thus, to approximate experimental loading conditions in the finite element model, force was applied to the pillar cap [Fig. S4(b)]. The fixed boundary condition (zero displacement) was prescribed at the bottom surface of the pillar. Pillar geometry was meshed using tetrahedral elements. Forces ranging from 0 to 200 *μ*N were applied, and pillar deformation was monitored. The slope of the force-displacement curve, i.e., pillar stiffness, was found to be 4.0 *μ*N/*μ*m.

### Immunofluorescence

To visualize muscle fibers and cross-striations, samples were fixed in 4% v/v paraformaldehyde in PBS for 4 h at 4 °C, permeabilized with 0.2% v/v Triton X-100 for 30 min at room temperature (RT), and then incubated in blocking buffer consisting of 5% v/v goat serum, 1% w/v bovine serum albumin, and 0.05% v/v Tween-20 in PBS (all reagents from Sigma) for 2 h at RT. Samples were then incubated overnight at 4 °C in rabbit anti-α-actinin (1:250, Abcam) primary antibody diluted in blocking buffer, followed by 2 h at RT in Alexa Fluor 488 goat-anti-rabbit IgG H&L (1:1000, Abcam) secondary antibody diluted in blocking buffer. Nuclei were stained with DAPI (5 *μ*g/ml, Invitrogen) for 30 min at RT. To visualize neuromuscular units, AChRs were labeled with Alexa Fluor 647-conjugated α-bungarotoxin (2 *μ*g/ml, Invitrogen) for 1 h at 37 °C, and samples were fixed, permeabilized, and blocked as before. Samples were then incubated overnight at 4 °C in rabbit anti-β-tubulin III (1:1000, Synaptic Systems) primary antibody diluted in blocking buffer, followed by 2 h at RT in Alexa Fluor 488 goat-anti-rabbit IgG H&L (1:1000, Abcam) secondary antibody diluted in blocking buffer. In all experiments, samples were rinsed 3 × 5 min with PBS between each incubation. Samples were embedded in ProLong Glass Antifade mountant (Invitrogen), covered with a glass coverslip, allowed 24 h at RT for the mountant to cure, and then imaged on a Zeiss LSM 710 confocal microscope.

### Conditioned medium

C2C12 was plated in tissue culture flasks, allowed to reach full confluency, and then cultured in muscle differentiation medium for 6 days with daily medium replacements. On day 6, muscle differentiation medium was aspirated, and cells were rinsed with PBS and incubated in 0.1 ml/cm^2^ of Advanced DMEM/F-12 and Neurobasal medium at a volume ratio of 1:1 supplemented with 2 mM L-glutamine (all from Gibco). After 24 h, this basal CM was collected, filtered using syringe filters with a pore size of 0.22 *μ*m, and neutralized to pH 7–7.4 using 1N sodium hydroxide. The full CM consists of basal CM, Advanced DMEM/F-12, and Neurobasal medium at a volume ratio of 2:1:1, with 10% v/v KnockOut serum replacement, 2 mM L-glutamine, 0.1 mM β-mercaptoethanol, and GDNF and CNTF at 10 ng/ml each. NDM with 10 ng/ml each of GDNF and CNTF was used as control.

### MEA preparation, recording, and data analysis

12-well MEA plates (Axion BioSystems) contained 64 embedded 30 *μ*m-diameter gold microelectrodes per well, spaced 200 *μ*m apart. Plates were prepared for cell seeding according to the manufacturer's protocols. Wells were coated with 0.1% polyethyleneimine for 1 h at 37 °C, then rinsed 3 times with PBS, and allowed to air dry in a biosafety cabinet overnight. The following day, wells were coated with 20 *μ*g/ml of laminin (Sigma) for 2 h at 37 °C before cell seeding. Neurospheres were dissociated in 0.05% w/v trypsin at 37 °C for ∼5 min and passed through a 40 *μ*m strainer to obtain a cell suspension. Dissociated cells were suspended in a 1:1 mixture of 20 *μ*g/ml laminin and NDM supplemented with 10 ng/ml GDNF and 10 ng/ml CNTF and seeded on MEA plates as a droplet centered over the electrode grid, at a density of 80 000 cells/well. Cells were incubated for 2 h at 37 °C to allow for attachment and then inundated in NDM supplemented with 10 ng/ml GDNF and 10 ng/ml CNTF. Plates received medium changes every other day. Electrical activity on the MEAs was recorded using the Maestro system and AxIS software (both from Axion BioSystems), using the following settings: bandpass filter (Butterworth, 300–5000 Hz), spike detector (adaptive threshold crossing, 8×SD of RMS noise), and burst detector (100 ms maximum inter-spike interval, five spikes minimum, ten spikes minimum for network bursts, and tens mean firing rate detection window). Recordings were performed daily for 15 min at 37 °C. Raw files were processed offline, skipping the first 5 min of each recording as an acclimation period. Raster plots were generated using the Neural Metric tool (Axion BioSystems).

## SUPPLEMENTARY MATERIAL

See the supplementary material for supplementary figures illustrating separate muscle tissue seeding (Fig. S1), time evolution of neural activity on MEAs (Fig. S2), schematic of the PDMS platform fabrication process (Fig. S3), and pillar stiffness calculation (Fig. S4) and supplementary movies showing muscle contractions of a co-culture sample (Movie S1) and spontaneous contractions of muscle strips from co-culture and muscle-only groups.

## References

[1] L. Ricotti , P. Dario , A. Menciassi , B. Trimmer , A. W. Feinberg , R. Raman , K. K. Parker , R. Bashir , M. Sitti , and S. Martel , Sci. Rob. 2, eaaq0495 (2017). 10.1126/scirobotics.aaq0495 33157905

[2] A. W. Feinberg , A. Feigel , S. Shevkoplyas , S. Sheehy , M. George , and K. K. Parker , Science 317, 1366 (2007). 10.1126/science.1146885 17823347

[3] J. C. Nawroth , H. Lee , A. W. Feinberg , C. M. Ripplinger , M. L. McCain , A. Grosberg , J. O. Dabiri , and K. K. Parker , Nat. Biotechnol. 30, 792 (2012). 10.1038/nbt.2269 22820316 PMC4026938

[4] V. Chan , K. Park , M. B. Collens , H. Kong , T. A. Saif , and R. Bashir , Sci. Rep. 2, 857 (2012). 10.1038/srep00857 23155480 PMC3498929

[5] B. J. Williams , S. V. Anand , J. Rajagopalan , and M. T. A. Saif , Nat. Commun. 5, 3081 (2014). 10.1038/ncomms4081 24435099

[6] S.-J. Park , M. Gazzola , K. S. Park , S. Park , V. Di Santo , E. L. Blevins , J. U. Lind , P. H. Campbell , S. Dauth , A. K. Capulli , F. S. Pasqualini , S. Ahn , A. Cho , H. Yuan , B. M. Maoz , R. Vijaykumar , J.-W. Choi , K. Deisseroth , G. V. Lauder , L. Mahadevan , and K. K. Parker , Science 353, 158 (2016). 10.1126/science.aaf4292 27387948 PMC5526330

[7] C. Cvetkovic , R. Raman , V. Chan , B. J. Williams , M. Tolish , P. Bajaj , M. S. Sakar , H. H. Asada , M. T. A. Saif , and R. Bashir , Proc. Natl. Acad. Sci. U. S. A. 111, 10125 (2014). 10.1073/pnas.1401577111 24982152 PMC4104884

[8] R. Raman , C. Cvetkovic , S. G. M. Uzel , R. J. Platt , P. Sengupta , R. D. Kamm , and R. Bashir , Proc. Natl. Acad. Sci. U. S. A. 113, 3497 (2016). 10.1073/pnas.1516139113 26976577 PMC4822586

[9] G. J. Pagan-Diaz , X. Zhang , L. Grant , Y. Kim , O. Aydin , C. Cvetkovic , E. Ko , E. Solomon , J. Hollis , H. Kong , T. Saif , M. Gazzola , and R. Bashir , Adv. Funct. Mater. 28, 1804580 (2018). 10.1002/adfm.201804580

[10] O. Aydin , X. Zhang , S. Nuethong , G. J. Pagan-Diaz , R. Bashir , M. Gazzola , and M. T. A. Saif , Proc. Natl. Acad. Sci. U. S. A. 116, 19841 (2019). 10.1073/pnas.1907051116 31527266 PMC6778261

[11] M. Knipper and R. J. Rylett , Neurochem. Int. 31, 659 (1997). 10.1016/S0197-0186(97)00009-0 9364452

[12] H. Wu , W. C. Xiong , and L. Mei , Development 137, 1017 (2010). 10.1242/dev.038711 20215342 PMC2835321

[13] Z. W. Hall and J. R. Sanes , Cell 72, 99 (1993). 10.1016/S0092-8674(05)80031-5 8428377

[14] N. Tabti and M. Poo , Prog. Brain Res. 84, 63 (1990). 10.1016/S0079-6123(08)60889-4 1980021

[15] O. E. Harish and M. Poo , Neuron 9, 1201 (1992). 10.1016/0896-6273(92)90077-Q 1334421

[16] M. A. Fox , J. R. Sanes , D.-B. Borza , V. P. Eswarakumar , R. Fässler , B. G. Hudson , S. W. M. John , Y. Ninomiya , V. Pedchenko , S. L. Pfaff , M. N. Rheault , Y. Sado , Y. Segal , M. J. Werle , and H. Umemori , Cell 129, 179 (2007). 10.1016/j.cell.2007.02.035 17418794

[17] S. Grillner , Nat. Rev. Neurosci. 4, 573 (2003). 10.1038/nrn1137 12838332

[18] S. Grillner , Neuron 52, 751 (2006). 10.1016/j.neuron.2006.11.008 17145498

[19] M. C. Tresch and O. Kiehn , Nat. Neurosci. 3, 593 (2000). 10.1038/75768 10816316

[20] Y. Morimoto , H. Onoe , and S. Takeuchi , Adv. Rob. 33, 208 (2019). 10.1080/01691864.2019.1567382

[21] Y. Shin , S. Han , J. S. Jeon , K. Yamamoto , I. K. Zervantonakis , R. Sudo , R. D. Kamm , and S. Chung , Nat. Protoc. 7, 1247 (2012). 10.1038/nprot.2012.051 22678430 PMC4035049

[22] J. Song , J. H. Shawky , Y. T. Kim , M. Hazar , P. R. LeDuc , M. Sitti , and L. A. Davidson , Biomaterials 58, 1 (2015). 10.1016/j.biomaterials.2015.04.021 25933063 PMC4437865

[23] S. G. M. Uzel , R. J. Platt , V. Subramanian , T. M. Pearl , C. J. Rowlands , V. Chan , L. A. Boyer , P. T. C. So , and R. D. Kamm , Sci. Adv. 2, e1501429 (2016). 10.1126/sciadv.1501429 27493991 PMC4972469

[24] C. E. Henderson , M. Huchet , and J.-P. Changeux , Proc. Natl. Acad. Sci. U. S. A. 78, 2625 (1981). 10.1073/pnas.78.4.2625 6941315 PMC319402

[25] E. E. Zahavi , A. Ionescu , S. Gluska , T. Gradus , K. Ben-Yaakov , and E. Perlson , J. Cell Sci. 128, 1241 (2015). 10.1242/jcs.167544 25632161 PMC4359927

[26] S. Chiron , C. Tomczak , A. Duperray , J. Lainé , G. Bonne , A. Eder , A. Hansen , T. Eschenhagen , C. Verdier , and C. Coirault , PLoS One 7, e36173 (2012). 10.1371/journal.pone.0036173 22558372 PMC3338613

[27] W. R. Legant , A. Pathak , M. T. Yang , V. S. Deshpande , R. M. McMeeking , and C. S. Chen , Proc. Natl. Acad. Sci. U. S. A. 106, 10097 (2009). 10.1073/pnas.0900174106 19541627 PMC2700905

[28] N. Rabieh , S. M. Ojovan , N. Shmoel , H. Erez , E. Maydan , and M. E. Spira , Sci. Rep. 6, 36498 (2016). 10.1038/srep36498 27812002 PMC5095645

[29] E. Bandi , A. Bernareggi , M. Grandolfo , C. Mozzetta , G. Augusti-Tocco , F. Ruzzier , and P. Lorenzon , J. Physiol. 568, 171 (2005). 10.1113/jphysiol.2005.091439 16037088 PMC1474771

[30] S. Thesleff , Int. Rev. Neurobiol. 28, 59 (1986). 10.1016/S0074-7742(08)60106-3 3026985

[31] S. Thesleff , Prog. Brain Res. 84, 93 (1990). 10.1016/S0079-6123(08)60892-4 2267320

[32] Y. I. Kim , T. Lomo , M. T. Lupa , and S. Thesleff , J. Physiol. 356, 587 (1984). 10.1113/jphysiol.1984.sp015484 6520797 PMC1193183

[33] Z.-P. Xie and M.-M. Poo , Proc. Natl. Acad. Sci. U. S. A. 83, 7069 (1986). 10.1073/pnas.83.18.7069 3462745 PMC386654

[34] Y. Kidokoro and M. Saito , Proc. Natl. Acad. Sci. U. S. A. 85, 1978 (1988). 10.1073/pnas.85.6.1978 3279423 PMC279905

[35] M. Weitkunat , M. Brasse , A. R. Bausch , and F. Schnorrer , Development 144, 1261 (2017). 10.1242/dev.140723 28174246 PMC5399620

[36] A. F. M. Johnstone , G. W. Gross , D. G. Weiss , O. H. U. Schroeder , A. Gramowski , and T. J. Shafer , Neurotoxicology 31, 331 (2010). 10.1016/j.neuro.2010.04.001 20399226

[37] E. Biffi , G. Regalia , A. Menegon , G. Ferrigno , and A. Pedrocchi , PLoS One 8, e83899 (2013). 10.1371/journal.pone.0083899 24386305 PMC3873984

[38] D. A. Wagenaar , J. Pine , and S. M. Potter , BMC Neurosci. 7, 11 (2006). 10.1186/1471-2202-7-11 16464257 PMC1420316

[39] S. Illes , W. Fleischer , M. Siebler , H. P. Hartung , and M. Dihné , Exp. Neurol. 207, 171 (2007). 10.1016/j.expneurol.2007.05.020 17644089

[40] T. J. Heikkilä , L. Ylä-Outinen , J. M. A. Tanskanen , R. S. Lappalainen , H. Skottman , R. Suuronen , J. E. Mikkonen , J. A. K. Hyttinen , and S. Narkilahti , Exp. Neurol. 218, 109 (2009). 10.1016/j.expneurol.2009.04.011 19393237

[41] H. Wichterle and M. Peljto , Curr. Protoc. Stem Cell Biol. 5, 1H.1.1 (2008). 10.1002/9780470151808.sc01h01s5 18770634

[42] T. Osaki , S. G. M. Uzel , and R. D. Kamm , Nat. Protoc. 15, 421 (2020). 10.1038/s41596-019-0248-1 31932771

